# Autism associated with 12q (12q24.31-q24.33) deletion: further report of an exceedingly rare disorder

**DOI:** 10.31744/einstein_journal/2020RC5335

**Published:** 2020-06-03

**Authors:** Jaime Lin, Gigliolle Romancini de Souza-Lin, Fernanda Coan Antunes, Letícia Burato Wessler, Emílio Luiz Streck, Cinara Ludvig Gonçalves

**Affiliations:** 1 Universidade do Sul de Santa Catarina TubarãoSC Brazil Universidade do Sul de Santa Catarina, Tubarão, SC, Brazil.; 2 Universidade do Extremo Sul Catarinense CriciúmaSC Brazil Universidade do Extremo Sul Catarinense, Criciúma, SC, Brazil.; 3 Hospital Nossa Senhora da Conceição TubarãoSC Brazil Hospital Nossa Senhora da Conceição, Tubarão, SC, Brazil.

**Keywords:** Chromosome aberrations, Nervous system malformations, Developmental disabilities, Autism spectrum disorder, 12q24.31 deletion syndrome

## Abstract

Chromosomal abnormalities are responsible for several congenital malformations in the world, some of these are associated to telomeric/subtelomeric deletions. The abnormalities involving the telomere of chromosome 12 are rare, with few reports of deletions involving 12q24.31 region in the literature, and, to our knowledge, only four of them in the 12q24.31-q24.33 region. We report a further case of interstitial deletion of bands 12q24.31-q24.33 associated with autism spectrum disorder. A 2-year-old boy with global developmental delay associated with multiple congenital anomalies. The Human Genome CGH Microarray 60K confirmed the diagnosis of 12q deletion syndrome. This study made a review of the current literature comparing our patient with previously reported cases. These detailed analyses contribute to the development of genotype/phenotype correlations for 12q deletions that will aid in better diagnosis and prognosis of this deletion.

## INTRODUCTION

Alteration of gene dosage due to gains or deletions of large genomic regions causes many genetic disorders, which are frequently associated with intellectual disability, autism spectrum disorders (ASD) and other phenotypic findings.^(1)^

Autism spectrum disorder is a set of neurodevelopmental disorders characterized by a *deficit* in social behaviors and nonverbal interactions, such as reduced eye contact, facial expression, and body gestures in the first 3 years of life. It is not a single disorder, and it is broadly considered to be a multi-factorial disorder resulting from genetic and non-genetic risk factors and their interaction. Although very complex and heterogenic, ASD is a strongly genetic disorder.^(1)^

Among the most commonly employed techniques to detect ASD susceptibility genes, comparative genomic hybridization (CGH) technology has been widely used in research studies and in clinical practice of ASD, in order to detect copy number variants (CNV) throughout the genome. Copy number variants represent a significant source of genetic variability and are responsible of disease susceptibility for several neurobehavioral phenotypes.^(2)^

Some genetic anomalies causing several congenital malformations are associated to telomeric/subtelomeric deletions. Among these, chromosome 12q telomeric/subtelomeric deletions are rare and only few patients have been reported previously presenting small interstitial deletion of bands 12q24.31-q24.33, with no other karyotypic abnormalities.^(3-6)^

We report the case of a patient with interstitial deletion of bands 12q24.31-q24.33 and review the current literature, comparing with previously reported cases, including other few cases of deletion involving 12q24.31 region.

## CASE REPORT

A 2 year-old boy presented to pediatric neurology investigation due to global developmental delay associated with multiple congenital anomalies.

The patient was born from the first pregnancy of healthy, consanguineous (parents are first-degree cousins). At the time of pregnancy, the mother was 24-year-old and the father, 36 years old. No history of previous miscarriages, no report of use of prescribed or over-the-counter medicines, and no history of exposure to possible teratogenic products during pregnancy.

The pregnancy was complicated by ventricle enlargement detect by ultrasound at 5 months of gestation. The patient was born preterm, gestational age of 7 months, cesarean delivery. Birth weight was 1,720g (<3^th^ centile), length was 39cm (<3^th^ centile) and occipitofrontal circumference (OFC) was 31cm (<3^th^ centile).

The patient was hospitalized four times at early infancy, twice due to pulmonary infection, once at 6 months of age for surgical removement of an extra toe on his left foot and for undescended testicles, and a last time, at 8 months, for a ventriculoperitoneal shunt surgery because of hydrocephalus.

Early global delay of developmental milestones was present. He kept the head up at the age of 12 months, sat at 16 months.

**Figure 1:**
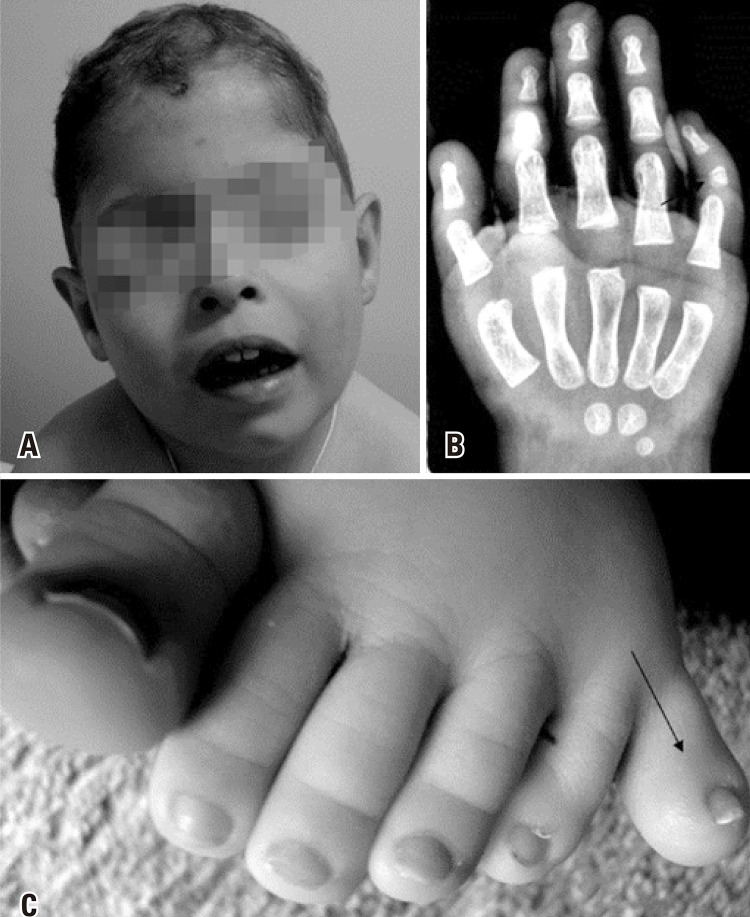
Multiple congenital anomalies. (A) Slight coarsening of facial appearance, a short nose with anteverted nares and smooth philtrum. The ears were normally set and normal in size and configuration; (B) Fifth finger clinodactyly on hand; (C) Polydactyly on the left foot

Currently, at 24 months, the patient presents failure to thrive, weight 10.6kg (5^th^ percentile); length 78cm (<5^th^ percentile) and OFC 48cm (50^th^ percentile). Physical features were significant for large anterior fontanelle and slight coarsening of facial appearance. He had a short nose with anteverted nares and smooth philtrum (Figure 1). The ears were normally set and normal in size and configuration. Palate was narrow with thick gums. The patient also presented fifth-finger clinodactyly and polydactyly on the left foot (Figure 1).

The neurological evaluation was noteworthy for severe agitation associated with a self-aggressive behavior characterized by head banging leading to severe self-inflicted injuries. The patient presented spastic hypertonus and brisk tendon reflexes, being unable to walk independently or speak.

**Figure 2:**
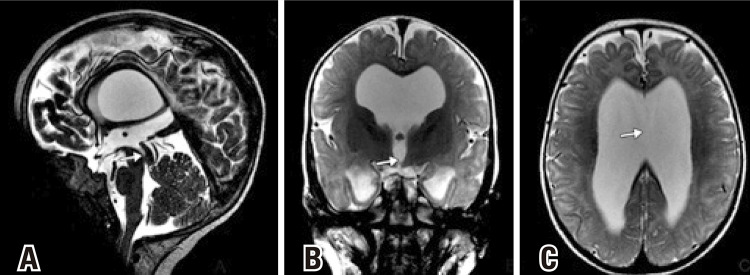
Magnetic resonance imaging T2-weighted sagital image. (A) Aqueductal web (arrow) with supratentorial hydrocephalus and ballooning of the chiasmatic recess. The corpus callosum was thin. Magnetic resonance imaging T2-weighted coronal image. (B) Axial image. (C) Dilatation of the lateral ventricles and of the third ventricle, septum pellucidum absence (arrow in C) and cerebral hypomyelination

Cranial magnetic resonance imaging revealed supratentorial hydrocephalus, ballooning of the chiasmatic recess, corpus callosum thin, dilatation of the lateral ventricles and of the third ventricle, absence of septum pellucidum, and cerebral hypomyelination (Figure 2).

The Human Genome CGH Microarray 60K (Agilent Technologies™) revealed an terminal deletion, starting from the middle of 12q24.31, between the genomic positions (123.309.075bp) to the near end of the q arm (132.283.607bp), arr[NCBI36/Hg18] 12q24.31-q24.33(123.309.075-132.283.607)X1, confirming the diagnosis of 12q deletion syndrome. The exam was performed in 2011 by the Human Genome Studies Center (Institute of Biosciences – *Universidade de São Paulo*).

## DISCUSSION

Technological advances in epidemiological and molecular genetics have led recently to new findings in the field of the genetics of neuropsychiatric disorders. These new findings concern also the domain of the genetics of autism and have broaden current knowledge on the genetic disorders associated with ASD.^(1)^

The number of known genetic disorders associated with ASD has increased with the use of array comparative genomic hybridization (aCGH). Such genetic diversity associated with similar autism cognitive-behavioral phenotypes created the concept of “syndromic autism” or “complex autism” (autism associated with genetic disorders/genetic syndromes), which qualifies individuals with at least one dysmorphic feature/malformation or severe intellectual disability. It is opposed to the concept of “non-syndromic autism” or “simplex”/“pure”/idiopatic autism (isolated autism) which qualifies individuals with moderate intellectual disability to normal cognitive functioning and no other associated signs or symptoms.^(2)^

The genetic investigation of such syndromic autism cases may lead to the recognition of rare and/or underreported diseases. Among these chromosome abnormalities, 12q24.31-q24.33 telomeric/subtelomeric deletions are rare and only a few patients have been reported previously.^(3)^Table 1 summarizes the main clinical findings of the previously reported cases.

**Table 1:** Clinical summary of cytogenetic abnormalities involving 12q telomere deletions

Genetic description	Reference	Phenotype
46, XY, del (12) (q 24.31-q24.33), mosaic	Al-Zahrani et al.^(3)^	8 years old, severe growth retardation with very low IGF-1 level, developmental retardation, dysmorphic face, low-set ears, cryptorchidism with short penis and elbow deformity
46, XY, 12q subtelomeric deletion; 1.6Mb; 14 genes	Niyazov et al.^(4)^	8 years old, delayed language development, food seeking behavior, attention deficit-hyperactivity disorder, high pain threshold, no facial dysmorphia, additional anterior second hair-whorl, brachydactyly, clinodactyly and obesity
46, XY, 12q subtelomeric deletion; 4.5Mb; 22 genes		12 years old, left cryptorchidism, renal abnormalities (multi-cystic left kidney, ectopic right kidney), epicanthal folds, small ears, delayed milestones, moderately ID, food seeking behavior and self-inflicting behavior
46, XY, del (12) (q24.31q24.32)	Plotner et al.^(5)^	9-months old, tracheomalacia, ambiguous genitalia; Dandy-Walker syndrome, coarse face, large anterior fontanella, short palpebral fissure, ptosis of the left eye, thick gums, large tongue, mild generalized hypotonia, bifid scrotum, bilaterally palpable testes, small penis, long fingers with patulous finger tips and developmental delay (at 9 months could not sit unsupported, nor crawled, nor babbled or cooed)
46, XY, del (12) (q24.31q24.33)	Sathya et al.^(6)^	12-months old, developmental delay (developmental level 10-12 months at 20 months of age), no genital abnormalities, microcephaly, large pubic fat pad, sacral pit, tapering fingers, clinodactyly, pes planovalgus, cardiac abnormalities (perimembranous VSD, mild tricuspid regurgitation, secundum ASD, mild biventricular and left atrial dilatation, mild tricuspid regurgitation), facial dysmorphia with large bulbous nose, smooth filtrum and large ears
46, XX, del (12) (q24.31)	Baple et al.^(7)^	13 years old, macroglossia, overgrowth, hyperinsulinism, developmental delay (walked at 18 months and was able to use a few words at 2 years), marked anxiety, autistic spectrum disorder, moderately to severely retarded, palpebral fissures, a broad nasal base, full cheeks, a highly arched palate, overcrowded teeth, full and everted lower lip, and large but narrow ears with a thick helix, inverted nipples, a single small truncal *café-au-lait* spot, large hands and feet, mild tapering of the fingers, and short toes, left 4th finger was proximally implanted, and shortening of the 4th metacarpal bone
46, XX, del (12) (q24.31)	Chouery et al.^(8)^	2 years old, global developmental delay (at 18 months of age, she was unable to sit without support, and she did not crawl, babble, her social interaction skills were lacking, and she rarely smiled in response, walking a few steps with support), infantile spasms, hypotonia, microcephaly, flat face, full cheeks, macroglossia, highly arched palate, retrognathia, narrow ear orifices, and *café-au-lait* spots, hypsarrhythmia
46, XX, del (12) (q24.31)	Qiao et al.^(9)^	8 years old, simplex ASD, global developmental delay, moderate ID, up-slanting palpebral fissures, synophrys, small, low-set and posteriorly rotated ears, high nasal root with thick alae nasi and a square tip, prominent front incisors and a narrow palate, very tapered fingers with prominent fingertip pads, bilateral hypoplastic nails on both halluces, patchy eczema and thick ichthyotic skin and diagnosed with a T-cell skin lymphoma (a hyperpigmented patch on her lower back)
46, XX, del (12) (q24.31)	Palumbo et al.^(10)^	12 years old, ID, seizures, stereotypies, macroglossia, full cheeks, persistent open foramen ovale, hypoglycemia episodes, spastic quadriplegia since the first months of life, global delay (starting to walk at 2.5 years and to speak at 4 years), hypoplastic nails with proximal implantation of the 4th metacarpal bone. Facial dysmorphic signs included long oval face, with downslanted palpebral fissures, and a broad nasal base with high nasal root, high palate with overcrowded teeth, full and everted lower lip vermilion, large and narrow ears with thick helix. A brain magnetic resonance imaging showed an ectasia of the ventricles
46, XY, del (12) (q24.31-q24.33) 9Mb	Present patient	12 years old, developmental retardation, unable to walk independently or speak, failure to thrive, large anterior fontanelle, coarse face with short nose with anteverted nares and smooth philtrum, high narrow palate with thick gums, fifth-finger clinodactyly on left hand, self-aggressive behavior, spastic hypertonus and brisk tendon reflexes

IGF-1: insulin-like growth factor-I; ID: intellectual disability; VSD: ventricular septal defect; ASD: atrial septal defect.

Our patient has some phenotypic findings that match from those presented in other patients with 12q deletions, as developmental delay, coarse face, growth failure, large anterior fontanella, delayed language development and clinodactyly. There was only one study indicating that parents were first cousins, which was also observed in the history of our patient. These findings detailed contribute to the development of genotype/phenotype correlations for 12q deletions and comparison with additional patients will continue to add to this clinical description.

The deleted region contains 52 annotated genes. Among these P2RX2 and ACADS are noteworthy for the developmental delay and behavioral and social problems presented in our patient.

## CONCLUSION

Detailed findings of rare syndromes contribute to the development of genotypic/phenotypic correlations for 12q deletions, and the comparison with additional patients will continue to add to this clinical description. Genetic investigation of cases of syndromic autism may lead to the recognition of rare and/or unreported diseases.

We emphasize the importance of genetic analysis for the investigation of chromosomal abnormalities in patients with intellectual disability, dysmorphism, developmental delay and multiple congenital anomalies.

## References

[1] 1. Park HR, Lee JM, Moon HE, Lee DS, Kim BN, Kim J, et al. A short review on the current understanding of autism spectrum disorders. Exp Neurobiol. 2016;25(1):1-13. Review. 10.5607/en.2016.25.1.1 PMC4766109 26924928

[2] 2. Marshall CR, Noor A, Vincent JB, Lionel AC, Feuk L, Skaug J, et al. Structural variation of chromosomes in autism spectrum disorder. Am J Hum Genet. 2008;82(2):477-88. 10.1016/j.ajhg.2007.12.009 PMC2426913 18252227

[3] 3. Al-Zahrani J, Al-Dosari N, AbuDheim N, Alshidi TA, Colak D, Al-Habit O, et al. Chromosome 12q24.31-q24.33 deletion causes multiple dysmorphic features and developmental delay: First mosaic patient and overview of the phenotype related to 12q24qter defects. Mol Cytogenet. 2011;4:9. 10.1186/1755-8166-4-9 PMC3083380 21457577

[4] 4. Niyazov DM, Nawaz Z, Justice AN, Toriello HV, Martin CL, Adam MP. Genotype/phenotype correlations in two patients with 12q subtelomere deletions. Am J Med Genet A. 2007;143A(22):2700-5. 10.1002/ajmg.a.32005 17937441

[5] 5. Plotner PL, Smith JL, Northrup H. Deletion 12q: a second patient with 12q24.31q24.32 deletion. Am J Med Genet A. 2003;118A(4):350-2. 10.1002/ajmg.a.10232 12687666

[6] 6. Sathya P, Tomkins DJ, Freeman V, Paes B, Nowaczyk MJ. De novo deletion 12q: report of a patient with 12q24.31q24.33 deletion. Am J Med Genet. 1999;84(2):116-9. 10.1002/(sici)1096-8628(19990521)84:2<116::aid-ajmg6>3.0.co;2-3 10323735

[7] 7. Baple E, Palmer R, Hennekam RC. A microdeletion at 12q24.31 can mimic beckwith wiedemann syndrome neonatally. Mol Syndromol. 2010;1(1):42-5. 10.1159/000275671 PMC2883851 20648245

[8] 8. Chouery E, Choucair N, Abou Ghoch J, El Sabbagh S, Corbani S, Mégarbané A. Report on a patient with a 12q24.31 microdeletion inherited from an insulin-dependent diabetes mellitus father. Mol Syndromol. 2013;4(3):136-42. 10.1159/000346473 PMC3638924 23653585

[9] 9. Qiao Y, Tyson C, Hrynchak M, Lopez-Rangel E, Hildebrand J, Martell S, et al. Clinical application of 2.7M Cytogenetics array for CNV detection in subjects with idiopathic autism and/or intellectual disability. Clin Genet. 2013; 83(2):145-54. 10.1111/j.1399-0004.2012.01860.x 22369279

[10] 10. Palumbo O, Palumbo P, Delvecchio M, Palladino T, Stallone R, Crisetti M, et al. Microdeletion of 12q24.31: report of a girl with intellectual disability, stereotypies, seizures and facial dysmorphisms. Am J Med Genet A. 2015; 167A(2):438-44. Review. 10.1002/ajmg.a.36872 25428890

