# Supplementary material for: Autism associated with 12q (12q24.31-q24.33) deletion: further report of an exceedingly rare disorder
Source: Einstein (Sao Paulo). 2020 Jun 3;18:eRC5335. doi: 10.31744/einstein_journal/2020RC5335 (PMC7279891; doi:10.31744/einstein_journal/2020RC5335)
Supplement: Translation

# Translation: Autismo associado à síndrome de deleção do cromossomo 12q24.31-q24.33: relato adicional de um distúrbio extremamente raro

**Authors:** Jaime Lin, Gigliolle Romancini de Souza-Lin, Fernanda Coan Antunes, Letícia Burato Wessler, Emílio Luiz Streck, Cinara Ludvig Gonçalves

**Affiliations:** 1 Brasil Universidade do Sul de Santa Catarina, Tubarão, SC, Brasil.; 2 Brasil Universidade do Extremo Sul Catarinense, Criciúma, SC, Brasil.; 3 Brasil Hospital Nossa Senhora da Conceição, Tubarão, SC, Brasil.

**Keywords:** Alterações cromossômicas, Malformações do sistema nervoso, Deficiências do desenvolvimento, Transtorno do espectro autista, Síndrome de deleção 12q24.31

## Abstract

Anomalias cromossômicas são responsáveis por inúmeras malformações congênitas no mundo, algumas delas associadas a deleções teloméricas/subteloméricas. As anomalias que envolvem o telômero do cromossomo 12 são raras, com poucos relatos na literatura sobre deleções relacionados à região 12q24.31 e, até onde sabemos, apenas quatro deles na região 12q24.31-q24.33. Relatamos um outro caso de deleção intersticial das bandas 12q24.31-q24.33 associada ao transtorno do espectro do autismo. Trata-se de um menino de 2 anos de idade com atraso global no desenvolvimento associado a múltiplas anomalias congênitas. A utilização do *Human Genome* CGH *Microarray* 60K confirmou o diagnóstico da síndrome de deleção 12q. Este estudo fez uma revisão da literatura atual, comparando nosso paciente com casos previamente relatados. Estas análises detalhadas contribuem para o desenvolvimento de correlações genótipo/fenótipo para deleções 12q, que ajudam aos melhores diagnóstico e prognóstico desta deleção.

## INTRODUÇÃO

A alteração da dosagem de genes devido a ganhos ou deleções de grandes regiões genômicas causa muitos transtornos genéticos, frequentemente associados a deficiência intelectual, Transtornos do Espectro Autista (TEA) e outros achados fenotípicos.^(1)^

O TEA é um conjunto de alterações do desenvolvimento neurológico, caracterizado por um *deficit* de comportamentos sociais e interações não verbais, como diminuição no contato visual, expressão facial e gestos corporais nos primeiros 3 anos de vida. Não se trata de um transtorno isolado, sendo geralmente considerado multifatorial, resultante de fatores de risco genéticos e não genéticos, e de sua interação. Embora bastante complexo e heterogênico, o TEA é um fortemente genético.^(1)^

Entre as técnicas mais utilizadas para detectar genes de suscetibilidade ao TEA, a tecnologia de hibridação genômica comparativa (CGH - *comparative genomic hybridization*) foi amplamente utilizada em estudos de pesquisa e na prática clínica de atendimento a TEA, a fim de detectar variantes no número de cópias (CNV - *copy number variants*) em todo o genoma. As variantes do número de cópias representam fonte significativa de variabilidade genética e são responsáveis pela suscetibilidade a doenças por vários fenótipos neurocomportamentais.^(2)^

Algumas anomalias genéticas que causam várias malformações congênitas estão associadas a deleções teloméricas/subteloméricas. Entre estas, são raras as deleções teloméricas/subteloméricas do cromossomo 12q, tendo sido relatados anteriormente uns poucos pacientes que apresentavam pequena deleção intersticial das bandas 12q24.31-q24.33, sem outras anormalidades cariotípicas.^(3-6)^

Relatamos o caso de um paciente com deleção intersticial das bandas 12q24.31-q24.33 e revisamos a literatura atual, comparando-o com casos relatados anteriormente, incluindo outros poucos casos de deleção envolvendo a região 12q24.31.

## RELATO DE CASO

Um menino de 2 anos de idade foi submetido à investigação neurológica pediátrica devido a atraso global no desenvolvimento associado a múltiplas anomalias congênitas.

O paciente nasceu da primeira gravidez de pais saudáveis e consanguíneos (os pais eram primos de primeiro grau). No momento da gravidez, a mãe estava com 24 anos, e o pai com 36 anos. Não havia história de abortos anteriores nem qualquer referência a uso de medicamentos com ou sem receita, e não havia história de exposição a possíveis produtos teratogênicos durante a gravidez.

A gravidez foi complicada por aumento ventricular detectado por ultrassom aos 5 meses de gestação. O paciente nasceu pré-termo aos 7 meses de idade gestacional por cesariana. O peso ao nascer foi de 1.720g (<3° percentil), a estatura era de 39cm (<3° percentil), e a circunferência occipitofrontal (COF) era de 31cm (<3° percentil).

O paciente foi hospitalizado quatro vezes na primeira infância, duas vezes por infecção pulmonar, uma vez aos 6 meses de idade para remoção cirúrgica de um dedo extra do pé esquerdo e testículos não descidos, e, na última vez, aos 8 meses, foi realizada derivação ventriculoperitoneal devido à hidrocefalia.

Apresentou atraso global precoce dos marcos do desenvolvimento. Sustentou a cabeça aos 12 meses e sentou-se aos 16 meses.

Atualmente, aos 24 meses de idade, o paciente apresenta *deficit* de crescimento, com peso de 10,6kg (5° percentil), estatura de 78cm (<5° percentil) e COF de 48cm (50° percentil). As características físicas de fontanela anterior grande são significativas, com leve embrutecimento das feições faciais. O paciente tinha nariz curto com narinas antevertidas e filtro liso (Figura 1). As orelhas tinham implantação, tamanho e configuração normais. O palato era estreito e alto, com gengivas grossas. O paciente também apresentava clinodactilia do quinto dedo e polidactilia no pé esquerdo (Figura 1).

**Figura 1:** Anomalias congênitas múltiplas. (A) Ligeiro embrutecimento das feições faciais, nariz curto com narinas antevertidas e filtro liso. As orelhas tinham implantação, tamanho e configuração normais; (B) Clinodactilia do quinto dedo da mão; (C) Polidactilia no pé esquerdo

Na avaliação neurológica, destacou-se agitação grave associada a um comportamento autoagressivo, caracterizado por pancadas na cabeça, resultando em lesões autoinfligidas graves. Apresentava hipertonia espástica e reflexos tendinosos vivos, sendo incapaz de andar de forma independente e de falar.

A ressonância magnética do crânio revelou hidrocefalia supratentorial, balonamento do recesso quiasmático, corpo caloso delgado, dilatação dos ventrículos laterais e do terceiro ventrículo, ausência de septo pelúcido e hipomielinização cerebral (Figura 2).

**Figura 2:** Imagem sagital ponderada em T2 da ressonância magnética. (A) Tela aqueductal (seta) com hidrocefalia supratentorial e balonamento do recesso quiasmático. O corpo caloso é delgado. Imagem coronal ponderada em T2 da ressonância magnética. (B) Imagem axial. (C) Dilatação dos ventrículos laterais e do terceiro ventrículo, ausência do septo pelúcido (seta em C) e hipomielinização cerebral

O *Human Genome* CGH *Microarray* 60K (Agilent Technologies™) revelou deleção terminal, começando no meio de 12q24.31, entre as posições genômicas (123.309.075bp) até a extremidade próxima do braço q (132.283.607bp), arr[NCBI36/Hg18] 12q24.31-q24.33 (123.309.075-132.283.607)X1, confirmando a síndrome de deleção 12q. O exame foi realizado em 2011 pelo Centro de Estudos do Genoma Humano (Instituto de Biociências – Universidade de São Paulo).

## DISCUSSÃO

Os avanços tecnológicos na genética epidemiológica e molecular levaram recentemente a novas descobertas no campo da genética dos transtornos neuropsiquiátricos. Essas novas descobertas dizem respeito também ao domínio da genética do autismo e ampliaram o estado atual do conhecimento sobre os transtornos genéticos associados ao TEA.^(1)^

O número de transtornos genéticos conhecidos associados ao TEA aumentou com o uso da hibridização genômica comparativa (aCGH). Essa diversidade genética associada a fenótipos cognitivo-comportamentais semelhantes ao autismo criou o conceito de “autismo sindrômico” ou “autismo complexo” (autismo associado a transtornos genéticos/síndromes genéticas), que qualifica indivíduos com pelo menos um aspecto dismórfico/malformação ou deficiência intelectual grave, opondo-se ao conceito de “autismo não sindrômico” ou “autismo simplex”/“puro”/idiopático (autismo isolado), que qualifica indivíduos que tenham desde uma deficiência intelectual moderada até um funcionamento cognitivo normal, sem outros sinais ou sintomas associados.^(2)^

A investigação genética desses casos de autismo sindrômico pode levar ao reconhecimento de doenças raras e/ou não relatadas. Entre essas anomalias cromossômicas, as deleções teloméricas/subteloméricas 12q24.31-q24.33 são raras, e apenas alguns pacientes foram relatados anteriormente.^(3)^ A tabela 1 resume os principais achados clínicos do casos relatados anteriormente.

**Tabela 1:** Resumo clínico de anormalidades citogenéticas envolvendo deleções de telômeros 12q

Descrição genética	Referência	Fenótipo
46, XY, del (12) (q 24.31-q24.33), mosaico	Al-Zahrani et al.^(3)^	8 anos de idade, retardo grave no crescimento, com nível muito baixo de IGF-1, retardo no desenvolvimento, face dismórfica, orelhas com baixa implantação, criptorquidia com pênis curto e deformidade do cotovelo
Deleção subtelomérica 46, XY, 12q; 1.6Mb; 14 genes	Niyazov et al.^(4)^	8 anos de idade, atraso no desenvolvimento da linguagem, comportamento de busca de alimentos, transtorno de *deficit* de atenção e hiperatividade, alto limiar de dor, sem dismorfia facial, segundo redemoinho capilar anterior adicional, braquidactilia, clinodactilia e obesidade
Deleção subtelomérica 46, XY, 12q; 4.5Mb; 22 genes		12 anos de idade, criptorquidia esquerda, anormalidades renais (rim esquerdo multicístico, rim direito ectópico), pregas epicânticas, orelhas pequenas, atraso nos marcos do desenvolvimento, DI moderada, comportamento de busca de alimentos e comportamento autoagressivo
46, XY, del (12) (q24.31q24.32)	Plotner et al.^(5)^	9 meses de idade, traqueomalácia, genitália ambígua; síndrome de Dandy-Walker, fácies grosseira, fontanela anterior grande, fissura palpebral curta, ptose do olho esquerdo, gengivas espessas, macroglossia, hipotonia generalizada leve, escroto bífido, testículos palpáveis bilateralmente, pênis pequeno, dedos longos com pontas patulosas e atraso no desenvolvimento (aos 9 meses não sentava sem apoio, não engatinhava, não balbuciava nem arrulhava)
46, XY, del (12) (q24.31q24.33)	Sathya et al.^(6)^	20 meses de idade, atraso no desenvolvimento (nível de desenvolvimento de 10-12 meses aos 20 meses de idade), sem anormalidades genitais, microcefalia, excesso de gordura pubiana, fosseta sacral, dedos afunilados, clinodactilia, pé plano valgo, anormalidades cardíacas (CIV - perimembranosa, insuficiência tricúspide leve, CIA tipo *ostium secundum*, dilatação atrial esquerda e biventricular leve), dismorfia facial com nariz bulboso grande, filtro liso e orelhas grandes
46, XX, del (12) (q24.31)	Baple et al.^(7)^	13 anos, macroglossia, crescimento excessivo, hiperinsulinismo, atraso no desenvolvimento (andou aos 18 meses e foi capaz de usar algumas palavras aos 2 anos), ansiedade acentuada, TEA, retardo moderado a grave, fissuras palpebrais, ampla base nasal, bochechas proeminentes, palato altamente arqueado, dentes apinhados, lábio inferior grosso e evertido, orelhas grandes, mas estreitas, com hélice espessa, mamilos invertidos, uma única mancha café-com-leite de pequena extensão no tronco, mãos e pés grandes, leve afunilamento dos dedos, dedos dos pés curtos, implantação proximal do quarto dedo e encurtamento do quarto osso metacarpo
46, XX, del (12) (q24.31)	Chouery et al.^(8)^	2 anos, atraso no desenvolvimento global (aos 18 meses de idade, não conseguia sentar-se sem apoio e não engatinhava nem balbuciava, carecia de habilidades de interação social, raramente sorria em resposta a estímulos, dava alguns passos com apoio), espasmos infantis, hipotonia, microcefalia, face plana, bochechas proeminentes, macroglossia, palato altamente arqueado, retrognatia, orifícios das orelhas estreitos, manchas café-com-leite e hipsarritmia
46, XX, del (12) (q24.31)	Qiao et al.^(9)^	8 anos, TEA simplex, atraso no desenvolvimento global, DI moderada, fissuras palpebrais oblíquas para cima, monocelha, orelhas pequenas, com implantação baixa e rotação posterior, raiz nasal alta com asas espessas e ponta quadrada, incisivos frontais proeminentes e palato estreito, dedos muito afunilados com pontas dos dedos proeminentes, unhas hipoplásicas nos dois hálux, eczema irregular e pele ictiótica espessa e diagnóstico de linfoma cutâneo de células T (mancha hiperpigmentada na região lombar)
46, XX, del (12) (q24.31)	Palumbo et al.^(10)^	12 anos de idade, DI, convulsões, estereotipias, macroglossia, bochechas proeminentes, forame oval patente, episódios de hipoglicemia, quadriplegia espástica desde os primeiros meses de vida, atraso global no desenvolvimento (começou a andar aos 2,5 anos e a falar aos 4 anos), unhas hipoplásicas com implantação proximal do 4º osso do metacarpo. Os sinais dismórficos faciais incluíam face oval longa, com fissuras palpebrais oblíquas, e uma base nasal ampla com raiz nasal alta, palato alto com dentes apinhados, vermelhão labial amplo e evertido, orelhas grandes e estreitas com hélice espessa. A ressonância magnética cerebral mostrou ectasia dos ventrículos
46, XY, del (12) (q24.31-q24.33) 9Mb	Paciente atual	2 anos de idade, retardo no desenvolvimento, incapaz de andar de forma independente ou de falar, falha de crescimento, fontanela anterior grande, fácies grosseira com nariz curto, narinas antevertidas e filtro liso, palato alto e estreito com gengivas grossas, clinodactilia do quinto dedo à esquerda, comportamento autoagressivo, hipertonia espástica e reflexos tendinosos hiperativos

IGF-1: fator de crescimento semelhante à insulina tipo 1; DI: deficiência intelectual; CIV: comunicação interventricular; CIA: comunicação interatrial; TEA: transtorno do espectro autista.

Nosso paciente tem alguns achados fenotípicos que coincidem com os apresentados em outros pacientes com deleções de 12q, como atraso no desenvolvimento, fácies grosseira, *deficit* de crescimento, fontanela anterior grande, atraso no desenvolvimento da linguagem e clinodactilia. Houve apenas um estudo indicando que os pais eram primos de primeiro grau, o que também foi observado na história de nosso paciente. Esses achados detalhados contribuem para o desenvolvimento de correlações genótipo/fenótipo para deleções de 12q, e a comparação com outros pacientes continua ampliando essa descrição clínica.

A região deletada contém 52 genes anotados. Entre esses, P2RX2 e ACADS se destacam pelo atraso no desenvolvimento e problemas comportamentais e sociais apresentados em nosso paciente.

## CONCLUSÃO

Achados detalhados de síndromes raras contribuem para o desenvolvimento de correlações genotípicas/fenotípicas para deleções de 12q, e a comparação com outros pacientes continuará a ampliar essa descrição clínica. A investigação genética de casos de autismo sindrômico pode levar ao reconhecimento de doenças raras e/ou não relatadas.

Enfatizamos a importância da análise genética para a investigação de anomalias cromossômicas em pacientes com deficiência intelectual, dismorfismo, atraso no desenvolvimento e múltiplas anomalias congênitas.
